# Development and Evaluation of Surveillance System for Identifying Jail-Associated COVID-19 Cases in Minnesota, USA, 2022

**DOI:** 10.3201/eid3013.230719

**Published:** 2024-04

**Authors:** Leah J. Porter, Erica Rapheal, Rebecca Huebsch, Tiana Bastian, Trisha J. Robinson, Hanna Chakoian, Karen G. Martin, Jennifer Zipprich

**Keywords:** COVID-19, 2019 novel coronavirus disease, coronavirus disease, severe acute respiratory syndrome coronavirus 2, SARS-CoV-2, viruses, respiratory infections, zoonoses, jails, correctional facilities, public health surveillance, residential facilities, electronic health records, electronic laboratory reports, Minnesota, United States

## Abstract

Confinement facilities are high-risk settings for the spread of infectious disease, necessitating timely surveillance to inform public health action. To identify jail-associated COVID-19 cases from electronic laboratory reports maintained in the Minnesota Electronic Disease Surveillance System (MEDSS), Minnesota, USA, the Minnesota Department of Health developed a surveillance system that used keyword and address matching (KAM). The KAM system used a SAS program (SAS Institute Inc., https://www.sas.com) and an automated program within MEDSS to identify confinement keywords and addresses. To evaluate KAM, we matched jail booking data from the Minnesota Statewide Supervision System by full name and birthdate to the MEDSS records of adults with COVID-19 for 2022. The KAM system identified 2,212 cases in persons detained in jail; sensitivity was 92.40% and specificity was 99.95%. The success of KAM demonstrates its potential to be applied to other diseases and congregate-living settings for real-time surveillance without added reporting burden.

Confinement facilities are high-risk settings for the spread of infectious diseases and were hotspots during the COVID-19 pandemic (*1*). Confinement facility design prioritizes security and space efficiency, creating inherent challenges to implementing disease mitigation strategies such as distancing, isolation, and quarantine (*1*–*3*). Spatial limitations can even disincentivize symptom reporting because of the use of solitary confinement spaces for medical isolation (*4*,*5*). Detained populations have limited autonomy to adopt prevention measures and are more vulnerable than the general population to severe disease resulting from higher rates of comorbidities and lower vaccine uptake (*6*–*10*). Frequent population turnover complicates contact tracing, generates continual infection introductions, and increases disease spread within and between confinement facilities (*3*,*11*). During the COVID-19 pandemic, US confinement facilities experienced increased staff turnover and strained staff capacity because of illness, especially during outbreaks (*12*,*13*).

The 82 jails in Minnesota, USA, are independently operated and vary greatly in size, technology, and healthcare infrastructure, unlike prisons, which are centrally operated (*14*). Jail capacity to implement COVID-19 mitigation strategies and deal with staffing shortages during outbreaks also varies widely between facilities (*15*). Rural and small jails in particular are more likely to have limited access to healthcare services and to lack electronic record systems because of funding constraints (*7*,*16*,*17*).

Public health practitioners at the Minnesota Department of Health (MDH) worked closely with confinement facility staff on COVID-19 surveillance and response, critical for ensuring access to testing, personal protective equipment, and therapeutics (*15*). In 2022, results of all professionally administered COVID-19 tests were reportable to the state as electronic laboratory reports (ELRs) and maintained in the Minnesota Electronic Disease Surveillance System (MEDSS). Therefore, confinement facilities were responsible for 2 types of COVID-19 public health reporting: ELRs for all tests they conducted (positive and negative results) and case reports for the persons working or detained in their facilities for whom test results were positive. 

Case reports alert public health authorities of diseases occurring in specific, high-risk settings (e.g., healthcare facilities and congregate settings including confinement, school, or childcare), and ELRs contain test results with identifying information for the patient, ordering provider, and performing laboratory. MEDSS used ELRs to create or update a person-level record that could be manually matched with a case report. However, there was no systematic method to associate MEDSS cases with confinement facilities from ELRs alone. MDH relied on facility-submitted case reports for situational awareness, and its ability to provide effective case response was affected by confinement facilities’ capacity for timely reporting.

In the fall of 2020, MDH staff began developing keyword and address matching (KAM) tools to identify COVID-19 cases among persons working or detained in confinement facilities directly from ELRs. By the fall of 2021, the complete system had been deployed, consisting of SAS code (SAS Institute Inc., https://www.sas.com) and an automated program within MEDSS that flagged cases for review by epidemiologists.

MDH used KAM to identify cases associated with all confinement settings, but the greatest effect was for jails. Cases associated with prisons were verified daily by using line lists from the Minnesota Department of Corrections. However, the same could not be done for jails because of the lack of a centralized testing and reporting system and varied reporting technology. We filled that gap by conducting a comprehensive evaluation of the KAM system for identifying COVID-19 cases among persons detained in jails.

## Methods

### KAM Surveillance System

The KAM surveillance system involved 2 steps. The first step was using KAM tools to search MEDSS ELR data and flag COVID-19 cases potentially associated with jails, and the second step was manually reviewing each case to verify confinement information and classify cases.

For step 1, a SAS program was used to clean addresses and phone numbers from ELR data and then identify records that matched any addresses or phone numbers of confinement facilities. The SAS program was later updated to identify keywords within addresses, case notes, and vaccination fields (Table 1). The SAS program was run on extracts of MEDSS data 2–5 times per week or as needed, depending on the daily volume of cases, and produced line lists of flagged cases for review in step 2.

**Table 1:** Keywords used by the Minnesota Department of Health to identify COVID-19 cases associated with confinement facilities, Minnesota, USA, 2022*

Keyword	Definition
Jail	
Correction	
Detention	
Prison	
ACF	Adult correction facility
ADC	Adult detention center
FCI	Federal correction institution
FPC	Federal prison camp
JDC	Juvenile detention center
JHS	Jail health services
Juvenile Center	
Juvenile Detention	
MCF	Minnesota correctional facility
Workhouse	
Intake	
DOC	Department of Corrections
Secure	
Work Release	
Reentry	
Sheriff	

*Blank cells indicate no definition necessary. †The keyword and address matching tools searched for variations of these keywords in several fields within COVID-19 electronic laboratory reports. Searches were case insensitive and allowed for variations in spacing and punctuation. Keywords listed refer to other types of confinement facilities but have removed those that identify individual facilities.

The automated keyword matching program within MEDSS was created to enable better coordination among MDH staff and more rapid responses. It updated every 15 minutes and had access to all ELRs associated with a COVID-19 case. The program could not perform address matching but searched for keywords within the ELR fields of ordering provider and performing laboratory. Records containing keywords were funneled into a MEDSS workflow for MDH staff to review.

For step 2, MDH staff reviewed each case flagged by KAM tools in step 1. They then compared them with public jail and prison rosters, case reports, previous ELRs, and other MEDSS records to verify dates of incarceration, facility information, and to determine the person’s case type (i.e., whether the person was detained or a staff member).

### KAM System Evaluation

**Figure 1:**
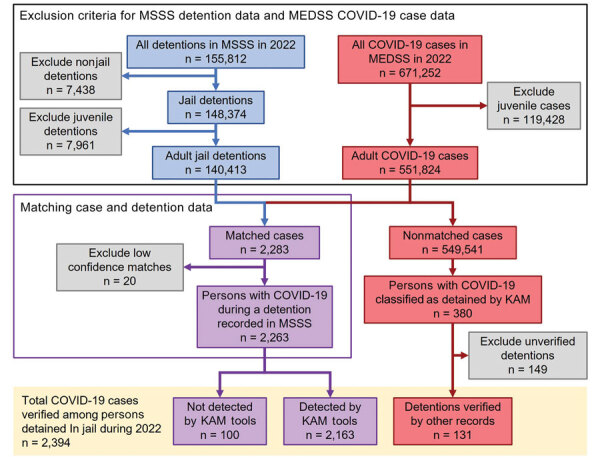
COVID-19 case data from MEDSS and jail detention data from MSSS. Exclusions were made before matching case and detention data by using an inexact matching threshold. A total of 380 unmatched cases had been flagged by KAM and classified as persons being detained in jail; further review verified 131 of those detentions, for a total of 2,394 COVID-19 cases among adults detained in jail. KAM, keyword and address matching; MDH, Minnesota Department of Health; MEDSS, Minnesota Electronic Disease Surveillance System; MSSS, Minnesota Statewide Supervision System; SAS, SAS Institute Inc., https://www.sas.com.

To evaluate the KAM system for detecting (step 1) and classifying (step 2) COVID-19 cases among persons detained in jail, we matched all COVID-19 cases in MEDSS that occurred in 2022 with jail detention data from the Minnesota Statewide Supervision System (MSSS). COVID-19 case data from MEDSS included full name, birthdate, specimen date, and confinement information documented by MDH staff during KAM step 2. Cases were included in this analysis if they met the criteria of a specimen date between January 1, 2022, and December 31, 2022, and if the person was >18 years of age at time of positive COVID-19 test (Figure 1).

With regard to jail detention data, MSSS is a statewide information system that stores data on persons who are or have been on probation, in detention, or imprisoned. Jails report these data to MSSS to create a centralized repository. Detention data include name, birthdate, detention dates, and facility name and were included in our analysis if they met the criteria of being detained during January 1, 2022–December 31, 2022; being detained in a Minnesota jail (excluding police departments and other nonjail facilities); and being >18 years of age at the time of detention (Figure 1).

We matched COVID-19 case data by full name and birthdate to MSSS detention data for persons with specimen dates that fell within their recorded detention period. We used an inexact match threshold to account for clerical errors, nicknames, and aliases. We manually reviewed low-confidence matches and subsequently excluded 20 records from analysis (Figure 1). Persons who were classified as jail detained by the KAM system but did not have an initial detention record match were reviewed for clerical errors and then rematched. For those remaining after the second match, we attempted to determine if their jail association could be verified with other records (e.g., criminal records and case investigation notes). We performed all analyses by using SAS 9.4.

## Results

### KAM System Results

**Figure 2:**
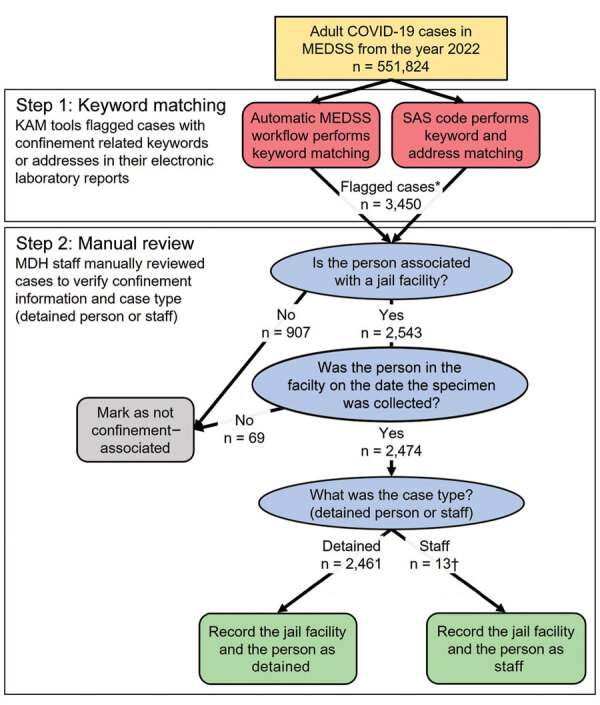
KAM surveillance system parts, process, and resulting unverified jail-associated COVID-19 case counts, Minnesota, USA, 2022 (cases among confirmed jail staff have been excluded from case counts). KAM consisted of KAM tools to flag COVID-19 cases potentially associated with jails and manual review to verify confinement information (e.g., facility name, dates incarcerated) and to classify the person as detained or facility staff (case type). Cases among persons confirmed to be jail staff have been excluded. Thirteen cases classified as staff were confirmed to have been for persons detained in jail by matching COVID-19 case data to detention data from MSSS. KAM, keyword and address matching; MEDSS, Minnesota Electronic Disease Surveillance System; MSSS, Minnesota Statewide Supervision System.

Throughout 2022, KAM step 1 (matching within MEDSS records) flagged 3,450 COVID-19 cases as being among persons potentially detained in jail (cases among known jail staff were excluded from this analysis). After manual review (KAM step 2), 2,461 (71%) persons were classified as detained at the time of their specimen collection (Figure 2).

### KAM Step 1 Evaluation Results

**Figure 3:**
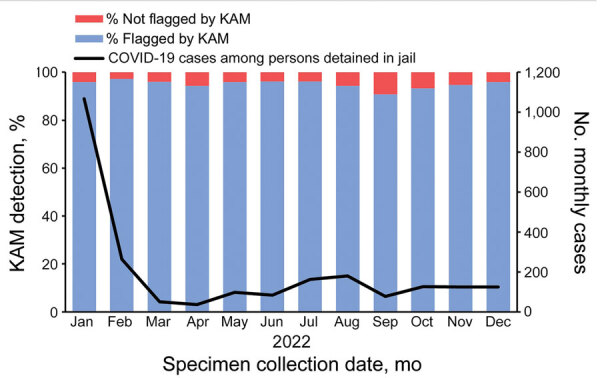
Monthly COVID-19 cases in persons detained in jail, Minnesota, USA, 2022, and the percentage detected by KAM surveillance tools and flagged for review. The figure includes 2,263 COVID-19 cases matched with a Minnesota Statewide Supervision System record of detention and 131 cases without a match that were confirmed as in persons detained at time of test (n = 2,394). KAM, keyword and address matching.

After excluding juvenile records, we included 551,824 COVID-19 cases from MEDSS and 140,413 detention records from MSSS in the matching analysis to match cases to detention records by full name and birthdate (Figure 1). After we excluded 20 low-confidence matches, 2,263 (0.4%) of the 551,824 COVID-19 cases had a detention documented in MSSS; 2,163 (95.6%) had been detected by KAM step 1, and 100 (4.4%) had not been detected. Of the 549,541 cases from MEDSS without a detention documented in MSSS, 380 (0.07%) had been classified by the KAM system as occurring in persons detained in jail. Further review of the records for those 380 persons confirmed that 131 (34.5%) of them were detained on the date of their specimen collection. In total, there were 2,394 COVID-19 cases among adults with verified detentions in a Minnesota jail in 2022. The matching tools in KAM step 1 had flagged 95.8% (2,163 + 131 = 2,294) of them (Figure 1). Despite substantial variance in case volume throughout the year, the monthly percentage of those cases flagged by KAM step 1 remained consistent (maximum 1,068 cases/month, 96% detected; minimum 36 cases/month, 94% detected) (Figure 3).

### KAM Step 2 Evaluation Results

Next, we evaluated the complete KAM surveillance system (steps 1 and 2). Throughout 2022, the KAM system detected and correctly classified 2,212 of 2,394 cases among adults detained in jail, for a sensitivity of 92.4%; it misclassified 249 of the 549,430 cases among adults not detained in jail, for a specificity of 99.95% (Table 2).

**Table 2:** Comparison of true detention status of persons with COVID-19 to the classifications made with KAM surveillance system, Minnesota, USA, 2022*

Classification	True detention status of persons with COVID-19†		
	Detained	Not detained	Total
Detained	2,212	249	2,461
Not detained	182	549,181	549,363
Total	2,394	549,430	551,824

*KAM, keyword and address matching. †Sensitivity 92.40%, specificity 99.95%.

Several factors contributed to false positives (false matches/classifications) and false negatives (missed matches/classifications) by the KAM system, including issues with detecting jail-associated cases in step 1 and misclassifications during the manual review process in step 2 (Table 3). More than half (n = 131) of false positives were attributed to persons previously detained but not in custody on the date of their specimen collection (however, 50% had positive test results within 7 days of intake or release); another 80 persons were jail staff misclassified as detained. Similarly, just under half of false negatives resulted from misclassifying persons detained in jail as not being jail associated (n = 69) or as jail staff members (n = 13). However, 100 cases (55.0% of false negatives) were not flagged by KAM in step 1.

**Table 3:** False-positive and false-negative classifications made by the KAM surveillance system while detecting and classifying COVID-19 cases among persons detained in jail, Minnesota, USA, 2022^*^

Category	No. (%) cases
False positives: KAM positive, true detention negative; 249 cases	
Detention did not overlap with specimen date	131 (52.6)
Jail staff mistaken as detained	80 (32.1)
Not jail-associated, erroneous identification in step 1†	38 (15.3)
False negatives: KAM negative, true detention positive; 182 cases	
Not identified in step 1†	100 (55.0)
Erroneously classified not jail-associated in step 2†	69 (37.9)
Detained person classified as jail staff	13 (7.1)

*KAM, keyword and address matching. †During KAM step 1, keyword and address matching tools flagged COVID-19 cases as potentially associated with jails; during step 2, Minnesota Department of Health staff manually reviewed each flagged case to verify confinement information and classify cases by jail facility and case type (detained person of staff) (Figure 2).

Last, 2,094 cases assigned to a jail during KAM step 2 matched with a detention record for comparison. Of those, ≈93% (n = 1,950) were recorded with the correct jail facility.

## Discussion

The sensitivity of the MDH KAM surveillance system for identifying COVID-19 cases among adults detained in Minnesota jails in 2022, without relying on case-based reporting from jails, was 92.4%. Despite KAM step 1 flagging ≈907 cases that were not jail-associated (Figure 2), manual record review during KAM step 2 contributed to an overall robust specificity for the surveillance system of 99.95%. Effective surveillance requires that cases can be associated with an individual facility, and the system was able to do this correctly 93.1% of the time.

Jurisdictional knowledge of jails and their testing practices was essential for determining the jail and case type (staff or detained person) of cases flagged by KAM tools. MDH staff used publicly available jail rosters, case reports from facilities, previous ELRs, and other MEDSS records for context, when available. Yet, interpreting ELRs can be complicated because of substantial variations by reporter and absence of case type indicators. In addition, ELRs may not include any confinement indicators if a person is tested outside the jail (e.g., hospital emergency room while in custody) or by a third-party vendor that does not indicate the ordering jail in the ELR. Most of the 69 cases erroneously marked as not jail-associated were in persons with a negative test result at the time of jail intake but who later had a positive test result while at an emergency department and still in custody. For those cases, keywords in ELRs for the previous intake tests were flagged, but the ordering provider and reporting laboratory (lack of keywords) in the ELR suggested that the person had been released from custody. Often, public detention data can clarify those situations, but they vary widely. Some jails do not publish any detention data, none publish data for youth, and many rosters list only current detentions, making retrospective review impossible. Timeliness and knowledge of local testing practices were often crucial for verifying a flagged case.

Keyword selection was also improved by jurisdictional knowledge and relationships. For example, many hospitals provided laboratory processing services to nearby jails and other institutions, which obscured the jail-associated tests. For one hospital, however, we learned that their billing department was already including an abbreviation in the ordering provider field, which we could also use for our purposes. Later, MDH coordinated with a state-sponsored testing vendor, which provided services to many facilities, to include keywords in their ELRs that would be identifiable and specific. The ability to easily update the KAM keywords enabled us to maintain real-time surveillance despite changes in testing vendors and reporting methods.

A weakness of the KAM system is that it relies on the assumption that all positive test results are promptly reported to the public health authority. Unreported results and over-the-counter test results (which do not produce ELRs) are undetectable without case-based reporting or other input. Jails may struggle most with reporting during an outbreak when staff capacity is most strained and reporting is arguably most important (*18*–*20*). Facilities operating with paper records or with limited on-site healthcare face additional challenges (*17*). Electronic health record systems can streamline documentation for reporting, benefiting the jail and the public health authority (*17*,*21*). Alternatively, outsourcing laboratory processing of tests transfers the reporting burden to the laboratory vendor. Maximizing the success of KAM may require supporting facilities in their reporting efforts.

Our assessment is limited by the nature of our input data, the quality of our matching process, and the inherent limitations of a retrospective study. MEDSS case data and MSSS detention data were vulnerable to clerical errors, aliases, and incomplete entries. Despite our accounting for some of those vulnerabilities with an inexact matching process, erroneous or missed matches in our dataset are possible. Our analysis identified 131 persons who had been detained but MSSS did not have a record of their detention (Table 4). Many were confirmed to have been held in jail by federal jurisdiction (i.e., the jurisdiction of the Federal Bureau of Prisons, Immigration and Customs Enforcement, or US Marshals), suggesting a systematic gap; however, most are probably accounted for by expected limitations in our datasets. Last, MDH did not keep thorough records of cases flagged by KAM that were not jail associated. Although we used a variable to denote cases as not confinement associated, the variable was used for functional purposes only and was probably not comprehensive; therefore, we cannot precisely quantify the total number of cases flagged in 2022.

**Table 4:** Results of investigating the 380 COVID-19 cases classified by KAM as persons detained in jail who did not have a detention recorded in MSSS for their specimen date, Minnesota, USA, 2022*

Category	No. (%) cases
Detention confirmed with other records	131 (34.5)
Specimen date outside of detention period	131 (34.5)
Jail staff misclassified as detained	80 (21.1)
Unable to determine	38 (10.0)

*KAM, keyword and address matching; MSSS, Minnesota Statewide Supervision System. †After matching COVID-19 case data with jail detention data, 380 cases remained that had been flagged by KAM tools during 2022 and classified as persons detained in jail. Those cases were individually reviewed to determine their correct classifications.

Infectious disease surveillance in jails is complex but essential, and KAM seems to be an effective tool for filling the gaps without increasing the reporting burden for the jail. MDH used KAM to provide situational awareness through early outbreak detection and case trends. It enabled public health practitioners to proactively connect with facilities experiencing new or continued outbreaks. MDH has also successfully applied KAM to identifying cases of COVID-19 among persons associated with homeless service sites, assisted living facilities, long-term care facilities, and higher education. Newer efforts have been successful in adapting KAM SAS code for other infectious diseases (e.g., group A *Streptococcus* in assisted living and long-term care facilities). We expect that those tools can be most successfully applied to other residential or congregate settings with somewhat stable populations or with on-site healthcare for diseases that are routinely tested for and reportable to public health authorities.
